# Exosomal miR-143-3p derived from follicular fluid promotes granulosa cell apoptosis by targeting BMPR1A in polycystic ovary syndrome

**DOI:** 10.1038/s41598-022-08423-6

**Published:** 2022-03-14

**Authors:** Yuanyuan Zhao, Shuhong Pan, Yunying Li, Xiaohua Wu

**Affiliations:** 1 Center for Reproductive Medicine, The Fourth Hospital of Shijiazhuang (Gynecology and Obstetrics Hospital Affiliated to Hebei Medical University), 206 East Zhongshan Road, Chang-An District, Shijiazhuang, 050011 Hebei China; 2 The Institute of Reproductive Health and Infertility, Shijiazhuang, 050011 Hebei China

**Keywords:** Endocrine system and metabolic diseases, miRNAs, Cell biology, Gene expression analysis, Reverse transcription polymerase chain reaction, Endocrine reproductive disorders

## Abstract

Polycystic ovary syndrome (PCOS) is an endocrine disorder that occurs in women of reproductive age. Anovulation caused by abnormal follicular development is still the main characteristic of PCOS patients with infertile. Granulosa cell (GC) is an important part of the follicular microenvironment, the dysfunction of which can affect follicular development. Increasing evidence indicates that exosomal miRNAs derived from the follicular fluid (FF) of patients play critical roles during PCOS. However, which follicular fluid-derived exosomal miRNAs play a pivotal role in controlling granulosa cell function and consequently follicular development remain largely unknown, as does the underlying mechanism. Herein, we showed that miR-143-3p is highly expressed in the follicular fluid exosomes of patients with PCOS and can be delivered into granulosa cells. Furthermore, functional experiments showed that translocated miR-143-3p promoted granulosa cell apoptosis, which is important in follicle development. Mechanistically, BMPR1A was identified as a direct target of miR-143-3p. Overexpression of BMPR1A reversed the effects of exosomal miR-143-3p on GC apoptosis and proliferation by activating the Smad1/5/8 signaling pathway. These results demonstrate that miR-143-3p-containing exosomes derived from PCOS follicular fluid promoted granulosa cell apoptosis by targeting BMPR1A and blocking the Smad1/5/8 signaling pathway. Our findings provide a novel mechanism underlying the roles of exosomal-miRNAs in the follicular fluid of PCOS patients and facilitate the development of therapeutic strategies for PCOS.

## Introduction

Polycystic ovary syndrome (PCOS) is an endocrine disorder in women of reproductive age, characterized by chronic anovulation, polycystic ovarian morphology and hyperandrogenism^1,2^. The etiology is complex, and the pathogenesis remains unclear. However, previous studies revealed that PCOS patients exhibit abnormal follicular development, which is the main cause of anovulatory infertility^3^.

The follicular microenvironment is essential for follicular development, oocyte maturation and quality^4^. It has been reported that the oocyte and surrounding granulosa cells (GCs) display an interdependent relationship in the stage of follicle development via direct gap junctions. It was also found that there was nutrient exchange and biological signal transmission between GCs and oocytes in the follicular fluid microenvironment through paracrine or autocrine manners, which regulated the growth, development and maturation of follicles^5,6^. Thus, the dysregulation of GCs affects the ovarian follicular microenvironment, which might impair folliculogenesis and subsequently lead to poor reproductive outcome in patients with PCOS. Therefore, exploring the function of GCs can explain the abnormal follicular development of patients with PCOS. Previous studies have reported that GC apoptosis affects follicle development, oocyte growth and maturation and triggers follicular atresia during the early phase of follicular development^7^. Increasing evidence indicates that the apoptosis of GCs in PCOS patients was significantly higher than that in healthy controls ^8,9^. However, the underlying mechanism of abnormal apoptosis of GCs involved in the process of abnormal follicular development in PCOS remains unclear.

Follicular fluid is the critical microenvironment for follicular development and contains a wide variety of biologically active molecules, including exosomes that have a lipid bilayer membrane structure with a diameter of 30–150 nm^10^. Exosomes contain cell-specific proteins, lipids, and nucleic acids that act as carriers passes biological information into target cells^11^. MicroRNAs (miRNAs) are small (21–24 nucleotides), single-stranded noncoding RNA molecules that can regulate target gene expression by binding to the 3’ untranslated regions (3’ UTRs) of target gene mRNAs to silence these genes at post transcriptional level^12^. A growing number of studies report that follicular fluid and exosomes derived from the follicular fluid of PCOS patients (PCOS-FF exosomes) have many differentially expressed nucleic acids, especially miRNAs^13–15^. However, the effect of FF-exosomal miRNAs on follicular development in patients with PCOS remains unclear.

In the present study, we mainly focused on exosomal-miR-143-3p and explored the effect of PCOS-FF derived exosomal-miR-143-3p on the apoptosis and proliferation of ovarian GCs and then analysed the underlying molecular mechanisms involved in follicular dysplasia in PCOS. Our findings will hopefully provide perspectives to understand the progression of PCOS.

## Results

### Comparison of clinical general information, laboratory data and apoptosis related indexes of granulosa cells between PCOS and normal controls

A total of 146 patients were enrolled in this study, including 80 healthy controls and 66 PCOS patients. As shown in Table 1, there were no substantial differences in patient age, infertility years, basal levels of E2 or prolactin (PRL) or days of stimulation between the two groups (p > 0.05). Body mass index (BMI), basal levels of LH and testosterone (T) and antral follicle count (AFC) in the PCOS group were significantly higher than those in the control group (p < 0.05). Additionally, the basal levels of FSH and the Gn dosage (IU) were significantly lower in the PCOS group than in the control group (p < 0.05). Laboratory data analysis indicated that the number of retrieved oocytes increased significantly, while the MII oocyte rate, 2PN fertilization rate and high-quality embryo rate in the PCOS group decreased significantly compared with those in the control group (p < 0.05). Consistently, we found that the pro-apoptotic gene Bax mRNA levels were significantly increased, and the anti-apoptotic gene Bcl-2 mRNA levels were significantly decreased in primary GCs in the PCOS group compared with the healthy control group (Supplementary Fig. S1).

**Table 1:** Comparison of clinical characteristics between healthy control group and PCOS group.

Variables	Healthy control group (n = 80)	PCOS group (n = 66)	*P* values
Age (years)	30.08 ± 3.42	29.05 ± 3.20	0.065
Body mass index (kg/m^2^)	23.30 ± 3.67	26.12 ± 4.21	0.000*
Infertility years (years)	2.59 ± 1.52	2.86 ± 1.74	0.317
Basal FSH (IU/L)	5.28 ± 1.60	4.72 ± 1.48	0.030*
Basal LH (IU/L)	3.61 ± 2.30	6.08 ± 5.52	0.001*
Basal E2 (pg/mL)	47.82 ± 44.99	45.90 ± 33.74	0.775
Basal PRL (μg/L)	14.75 ± 10.71	13.76 ± 7.18	0.519
Basal T (nmol/L)	1.01 ± 0.31	1.44 ± 0.48	0.000*
Antral follicle count (n)	18.33 ± 7.53	28.94 ± 13.34	0.000*
Days of stimulation (days)	12.31 ± 2.30	11.56 ± 2.60	0.066
Gn dosage (IU)	2613.96 ± 675.51	2316.11 ± 747.21	0.013*
Number of retrieved oocytes (n)	16.83 ± 6.02	19.18 ± 6.07	0.020*
MII oocytes rate (%)	92.51 ± 8.04	89.33 ± 11.09	0.047*
2PN fertilization rate (%)	74.39 ± 13.49	68.69 ± 16.44	0.023*
High-quality embryos rate (%)	54.19 ± 26.01	41.37 ± 19.64	0.001*

The data are presented as the mean ± SD.

*FSH* follicle-stimulating hormone, *E2* estradiol, *LH* luteinizing hormone, *PRL* prolactin, *T* testosterone, *MII* metaphase II oocytes.

Student’s t-test was used for statistical analysis *P < 0.05.

### Isolation and characterization of follicular fluid-derived exosomes

**Figure 1:**
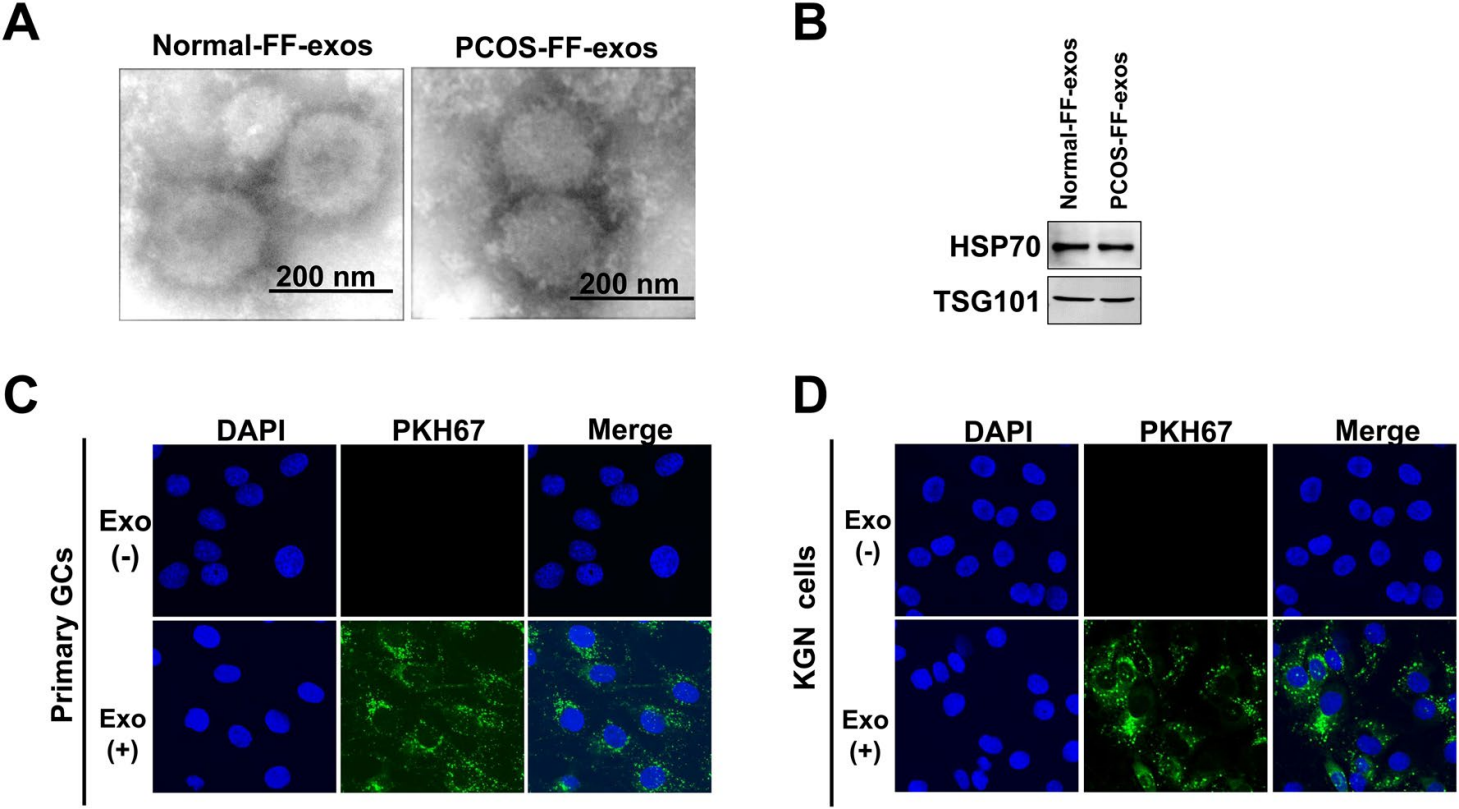
Isolation and identification of exosomes derived from human follicular fluids. **(A)** Transmission electron microscopy (TEM) analysis of the ultrastructure of exosomes from the FF of the normal population (normal-FF-exos) and the exosomes from the FF of patients with PCOS (PCOS-FF-exos). Scale bars, 200 nm. **(B)** Western blotting analysis of the exosome markers HSP70 and TSG101. Original blots/gels are presented in Supplementary Fig. S3. **(C)** Detection of FF-exosome uptake by primary GCs or KGN cells in vitro. Granulosa cells were incubated with PKH67-labeled exosomes for 12 h (PKH67 is shown in green, nuclei were stained with DAPI).

Exosomes derived from the FF of healthy control group and patients with PCOS were isolated and characterized. TEM analysis revealed that exosomes derived from the FF of patients with PCOS were round membrane-bound vesicles with a diameter of 30–150 nm, which was consistent with exosomes from controls (Fig. 1A). Western blotting showed that the exosome marker proteins TSG101 and HSP70 were enriched in these exosomes, as expected (Fig. 1B). To further confirm whether FF-exosomes can be taken up by primary GCs or KGN cells, we labeled the FF-exosomes with PKH67 fluorescent dye and then coincubated them with primary GCs or KGN cells for 12 h. Immunofluorescence staining revealed that the PKH67-labeled exosomes were efficiently absorbed by primary GCs or KGN cells and transferred to the cytoplasmic compartment (Fig. 1C,D). Overall, these results indicate that FF-exosomes can be successfully isolated and internalized by primary GCs or KGN cells.

### PCOS-FF derived exosomes promote granulosa cell apoptosis and inhibit proliferation in vitro

**Figure 2:**
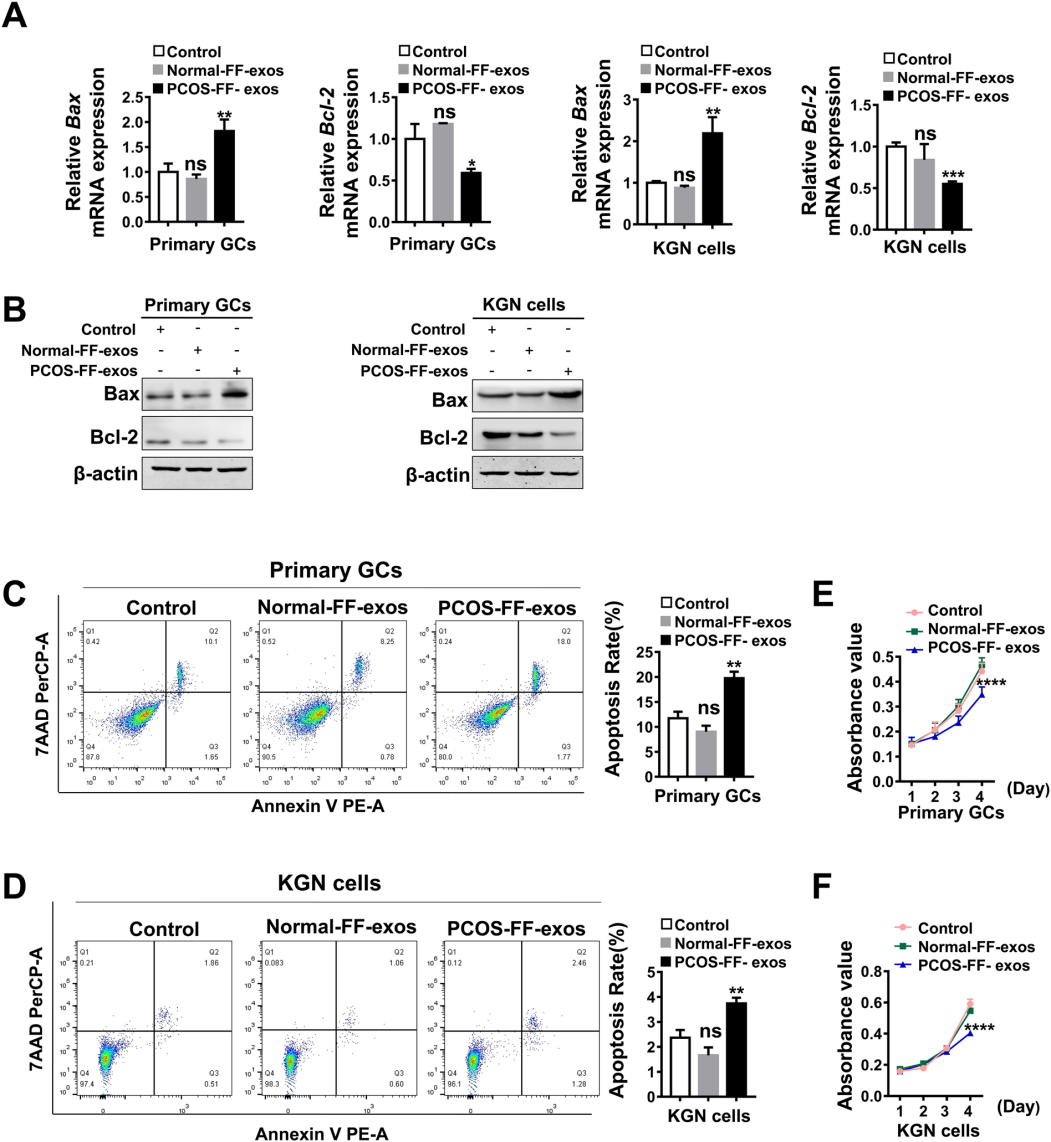
PCOS-FF exosomes accelerate granulosa cell apoptosis and reduce cell proliferation compared with normal-FF exosomes. **(A)** Quantitative PCR analysis of the expression levels of apoptosis-related genes in primary granulosa cells and KGN cell lines treated with normal-FF-exos or PCOS-FF-exos for the indicated times. **(B)** Western blotting analysis of the expression levels of apoptosis-related proteins in primary granulosa cells and KGN cell lines treated with normal-FF-exos or PCOS-FF-exos for the indicated times. Original blots/gels are presented in Supplementary Fig. S4. **(C,D)** Flow cytometry detection of apoptosis after primary granulosa cells and KGN cells were incubated with normal-FF-exos or PCOS-FF-exos for 48 h. **(E,F)** CCK8 analysis of cell viability after primary granulosa cells and KGN cell incubation with normal-FF-exos or PCOS-FF-exos for 3 days. All data are presented as the mean ± SD. Student’s t-test was used for statistical analysis. *ns* no significance, *P < 0.05, **P < 0.01, ***P < 0.001, ****P < 0.0001 for comparison with control.

To determine the functional role of PCOS-FF derived exosomes on GC apoptosis and proliferation, we cultured primary granulosa cells and KGN cells with normal-FF derived exosomes and PCOS-FF derived exosomes for 48 h, respectively. As shown in Fig. 2A,B, the mRNA and protein expression levels of the pro-apoptosis gene Bax were upregulated in GCs exposed to PCOS-FF derived exosomes, while the mRNA and protein expression levels of the anti-apoptosis gene Bcl-2 were downregulated in GCs exposed to PCOS-FF derived exosomes. As expected, the apoptotic rates of primary GCs and KGN cells were significantly enhanced in the PCOS-FF derived exosome- treated group, as determined by PE Annexin V assay (Fig. 2C,D). We further evaluated the effects of PCOS-FF derived exosomes on granulosa cell proliferation in vitro via a CCK8 assay. PCOS-FF derived exosome treatment significantly inhibited the growth capacity of primary GCs and KGN cells compared with PBS and normal-FF derived exosome treatment (Fig. 2E,F). Taken together, our findings show that PCOS-FF derived exosomes promote apoptosis and inhibit the proliferation of granulosa cells.

### miR-143-3p is enriched in PCOS-FF derived exosomes but not in normal-FF derived exosomes

**Figure 3:**
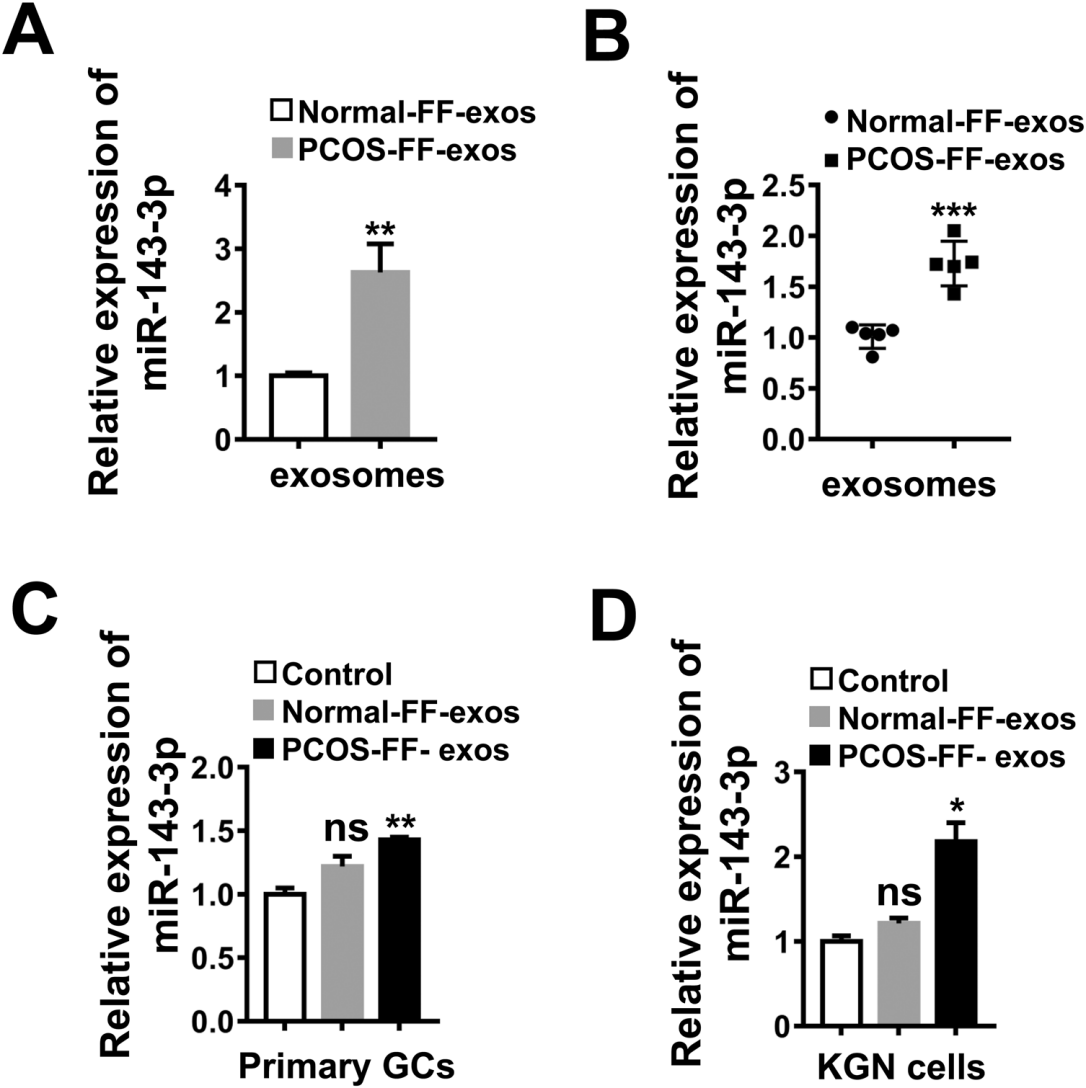
miR-143-3p is enriched in PCOS-FF derived exosomes but not in normal-FF derived exosomes. **(A)** *GRA001999* database analysis of miR-143-3p expression in PCOS-FF-exos. **(B)** Quantitative PCR analysis of the expression levels of miR-143-3p in normal-FF-exos and PCOS-FF-exos (n = 5). **(C,D)** Quantitative PCR analysis of the expression levels of miR-143-3p in primary granulosa cells and KGN cells after normal-FF-exos or PCOS-FF-exos treatment for 48 h. All data are presented as the mean ± SD. Student’s t-test was used for statistical analysis. *ns* no significance, *P < 0.05, **P < 0.01, ***P < 0.001, ****P < 0.0001.

Previous studies have shown that serum, follicular fluid and granulosa cells release large amounts of microvesicles containing both coding and noncoding RNAs, including miRNAs with multiple functional properties^16–19^. Thus, we hypothesized that PCOS-FF-exosomes might enhance apoptosis of granulosa cells by transferring specific miRNAs. Hence, we screened the differentially expressed miRNAs in follicular fluid derived exosomes between the normal population and patients with PCOS according to the *GRA001999* database of the National Genomics Data Center and found that miR-143-3p was enriched in exosomes of PCOS-FF, and the result was confirmed by the expression levels of miR-143-3p in normal-FF-exosomes and PCOS-FF-exosomes as detected by qRT–PCR analysis (Fig. 3A,B). In addition, miR-143-3p levels were significantly increased in primary granulosa cells and KGN cells treated with PCOS-FF- exosomes compared with PBS and normal-FF- exosomes (Fig. 3C,D). Thus, our results suggest that miR-143-3p is enriched in PCOS-FF derived exosomes.

### Exosomal-miR-143-3p promotes granulosa cell apoptosis and suppresses proliferation in vitro

**Figure 4:**
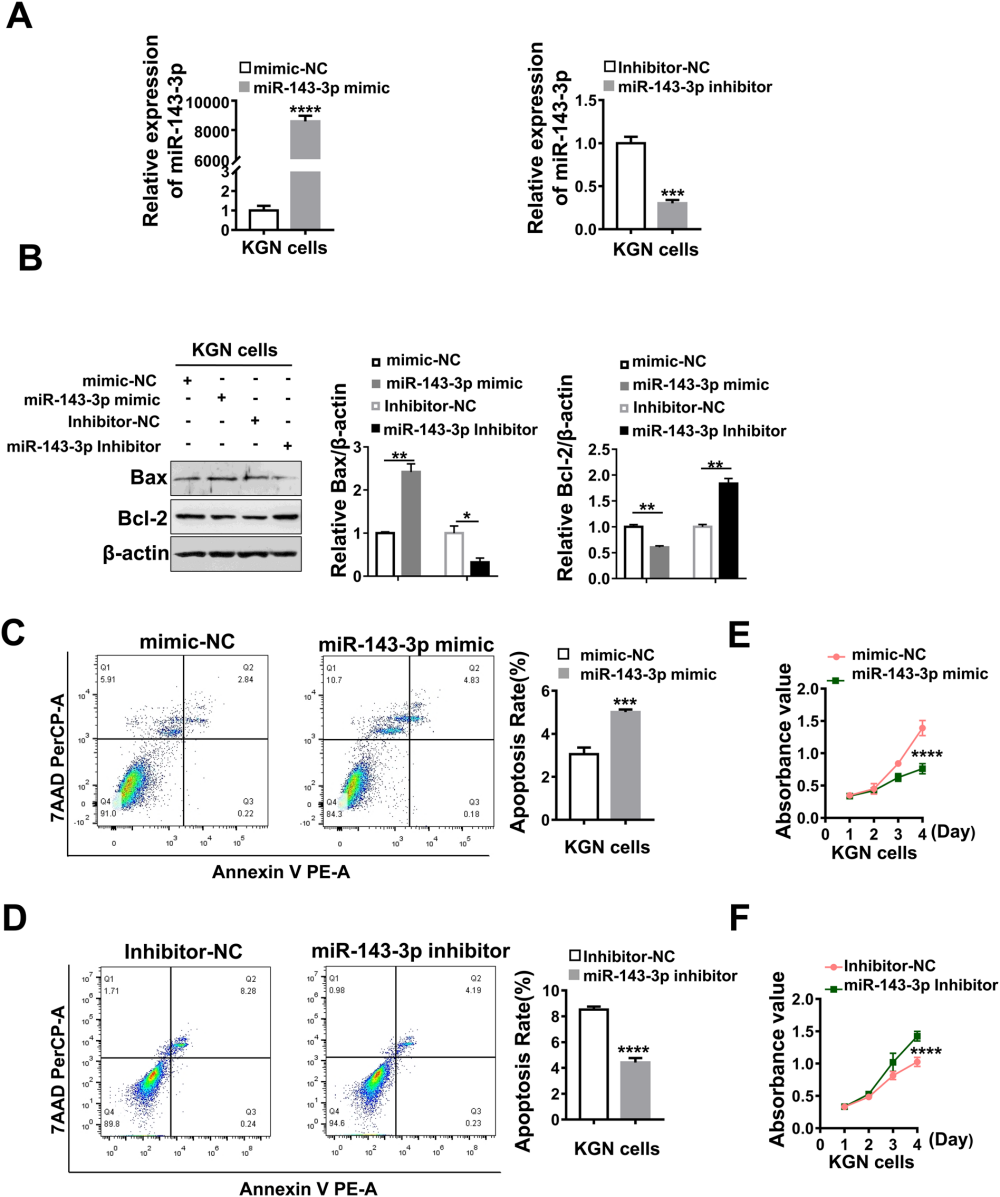
Exosomal-miR-143-3p promotes apoptosis and inhibits growth in KGN cells. **(A)** Quantitative PCR analysis of the expression levels of miR-143-3p in KGN cells transfected with miR-143-3p mimic or miR-143-3p inhibitor for 24 h. **(B)** Western blotting analysis of the expression levels of apoptosis-related proteins in KGN cells treated with miR-143-3p mimic or miR-143-3p inhibitor for 48 h. The protein expressions were quantified by using gray scale analysis. Original blots/gels are presented in Supplementary Fig. S5. **(C,D)** Flow cytometry detection of apoptosis after KGN cells were incubated with miR-143-3p mimic or inhibitor for 48 h. **(E,F)** CCK8 analysis of cell viability after primary granulosa cells and KGN cell incubation with miR-143-3p mimic or inhibitor for three days. All data are presented as the mean ± SD. Student’s t-test was used for statistical analysis. *ns* no significance, *P < 0.05, **P < 0.01, ***P < 0.001, ****P < 0.0001.

To further clarify the biological roles of miR-143-3p in granulosa cell apoptosis and proliferation, miR-143-3p mimic or inhibitor were transfected into KGN cells. As expected, miR-143-3p mimic significantly increased miR-143-3p expression, while miR-143-3p inhibitor decreased its expression in KGN cells (Fig. 4A). Western blotting results revealed that the protein levels of the pro-apoptotic gene Bax were significantly increased by miR-143-3p mimic transfection and decreased by miR-143-3p inhibitor transfection. However, the protein levels of the anti-apoptosis gene Bcl-2 were significantly decreased by miR-143-3p mimic transfection and increased by miR-143-3p inhibitor transfection. (Fig. 4B). Cell apoptosis assays showed that miR-143-3p overexpression significantly promoted cell apoptosis, whereas miR-143-3p inhibition showed an anti-apoptotic phenotype (Fig. 4C,D). Additionally, the CCK8 assay showed that miR-143-3p overexpression significantly suppressed cell proliferation, while miR-143-3p inhibition enhanced cell proliferation (Fig. 4E,F). These results reveal that PCOS-FF derived exosomal miR-143-3p plays a crucial role in enhancing granulosa cell apoptosis and reducing proliferation.

### BMPR1A is a direct target gene of miR-143-3p

**Figure 5:**
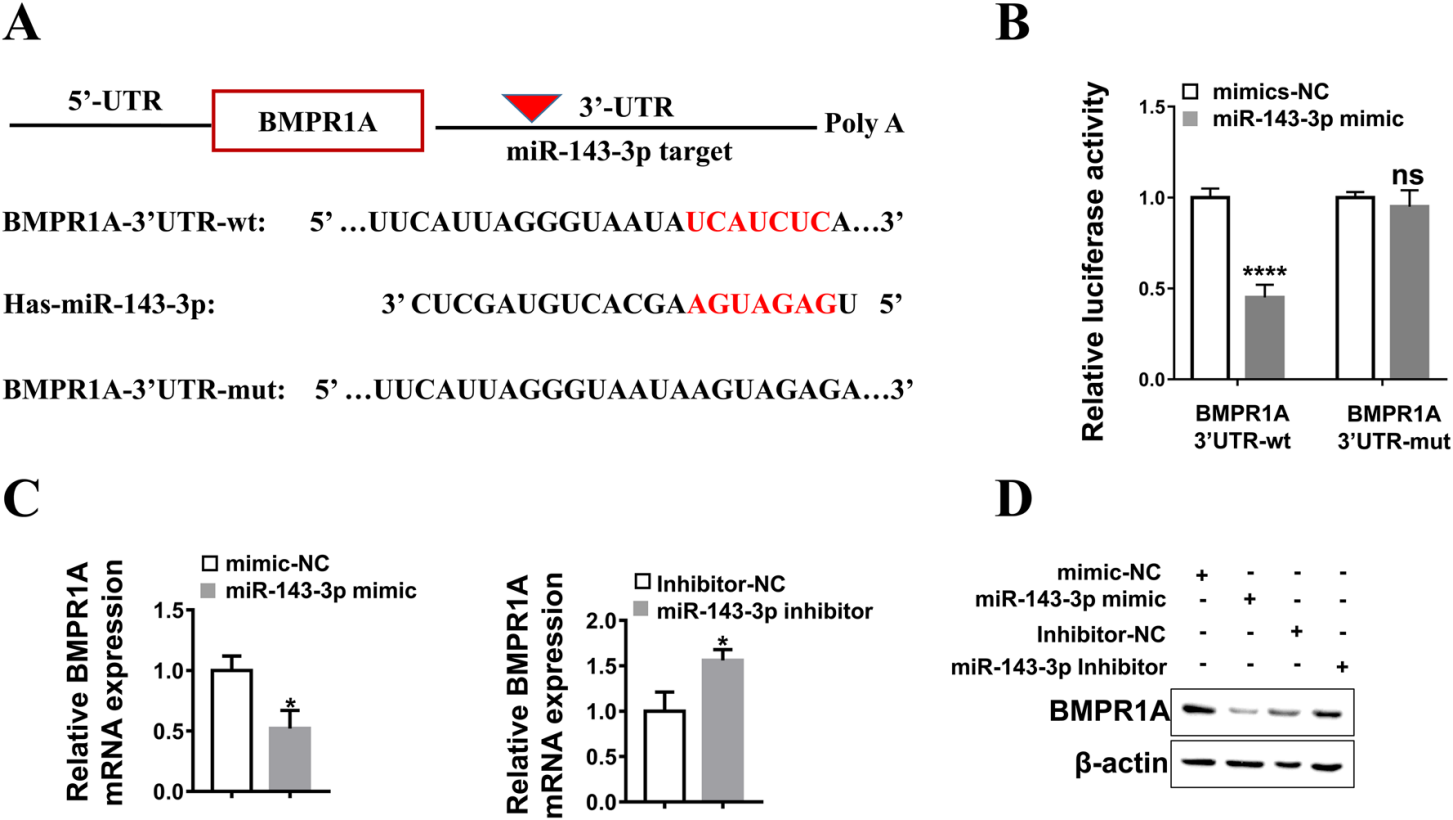
BMPR1A is a direct target gene of miR-143-3p. **(A)** The binding site of miR-143-3p on the BMPR1A gene was predicted by TargetScan Human 7.2. The wild-type BMPR1A-3’UTR (BMPR1A-3’UTR-wt) and mutant BMPR1A-3’UTR (BMPR1A-3’UTR-mut) were used to construct recombinant plasmids for dual luciferase reporter assays. wt, wild type; mut, mutant; UTR: untranslated region. **(B)** The relative luciferase activity was measured following cotransfection of miR-143-3p mimic with plasmids encoding BMPR1A-3’UTR-wt or BMPR1A-3’UTR-mut into KGN cells. The Renilla luciferase activities were normalized to firefly luciferase activities. **(C)** Quantitative PCR analysis of BMPR1A mRNA expression in KGN cells transfected with miR-143-3p mimic or inhibitor. **(D)** Western blotting analysis of BMPR1A protein expression in KGN cells transfected with miR-143-3p mimic or inhibitor. Original blots/gels are presented in Supplementary Fig. S6. *ns* no significance, *P < 0.05, **P < 0.01, ***P < 0.001. All results are presented as the mean ± SD.

To further elucidate the mechanism underlying miR-143-3p affecting granulosa cell function, we searched for miR-143-3p target genes using online bioinformatics prediction software (TargetScan Human7.2). The results demonstrated that a binding sequence of miR-143-3p was found in the 3’UTR of BMPR1A (Fig. 5A). BMPR1A is a bone morphogenetic protein receptor activin-like kinase and a key signaling molecule in the BMP signaling pathway that plays a key role in the cell cycle, proliferation, apoptosis and other cell processes^20,21^. Thus, we speculated that BMPR1A may be a direct target gene of miR-143-3p. We detected the mRNA expression of BMPR1A in GCs between control and PCOS patients, the result showed that the mRNA expression of BMPR1A in primary GCs of patients with PCOS were lower than in controls (Supplementary Fig. S2). Subsequently, a dual-luciferase reporter gene assay was applied to verify their interaction. PGL-3-BMPR1A-wt plasmids and PGL-3-BMPR1A-mut plasmids were constructed and cotransfected with miR-143-3p mimic into KGN cells. The results showed that luciferase activity was reduced in KGN cells cotransfected with miR-143-3p mimic and wild-type BMPR1A 3’UTR reporter, while no luciferase activity change was found in KGN cells cotransfected with miR-143-3p mimic and mutant BMPR1A 3’UTR reporter (Fig. 5B).

To investigate the relationship between BMPR1A and miR-143-3p, we examined BMPR1A mRNA and protein expression in KGN cells transfected with the miR-143-3p mimic or inhibitor. As shown in Fig. 5C,D, the mRNA and protein expression of BMPR1A were decreased by miR-143-3p mimic transfection and increased by miR-143-3p inhibitor transfection. These data indicate that BMPR1A is a direct target of miR-143-3p.

### BMPR1A overexpression attenuates the effects of miR-143-3p on cell apoptosis and proliferation in KGN cells

**Figure 6:**
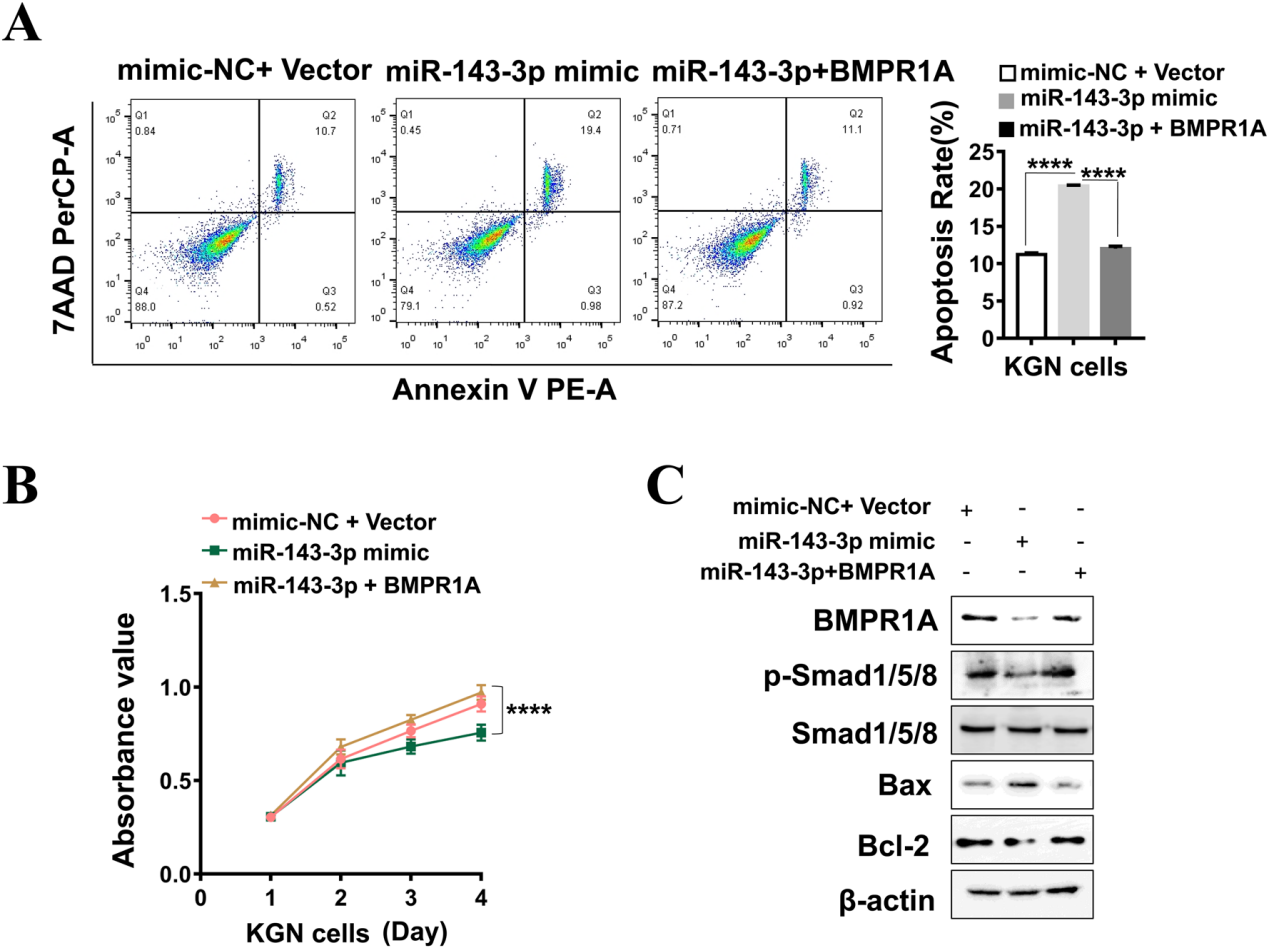
miR-143-3p promotes cell apoptosis and suppresses cell proliferation by inhibiting the BMPR1A/Smad1/5/8 signaling pathway. **(A)** Flow cytometry detection of apoptosis after KGN cells were cotreated with BMPR1A-expressing plasmid and miR-143-3p mimic. **(B)** CCK8 analysis of cell viability after KGN cells were cotreated with BMPR1A-expressing plasmid and miR-143-3p mimic. **(C)** Western blotting analysis showing the expression of BMPR1A, Smad1/5/8, p-Smad1/5/8 and apoptosis-related proteins in KGN cells treated with miR-143-3p mimic and/or BMPR1A-expressing plasmid. Original blots/gels are presented in Supplementary Fig. S7.* ns* no significance, *P < 0.05, **P < 0.01, ***P < 0.001. All results are presented as the mean ± SD.

To explore whether BMPR1A was involved in the regulation of miR-143-3p on cell phenotype, we cotransfected miR-143-3p mimic and pcDNA-BMPR1A plasmid in KGN cells. Cell apoptosis and CCK8 assays indicated that the combined transfection of miR-143-3p mimic and BMPR1A expression plasmid inhibited cell apoptosis and promoted cell proliferation compared with miR-143-3p mimic transfection alone (Fig. 6A,B). These data suggest that BMPR1A mediates the role of miR-143-3p in promoting cell apoptosis and inhibiting cell proliferation in KGN cells.

### The Smad1/5/8 signaling pathway may be involved in the regulation of exosomal miR-143-3p on cell apoptosis and proliferation

Previous studies have shown that Smad1/5/8 are BMPR1A downstream signaling molecules that regulate cell proliferation and apoptosis^22^. Next, we detected whether the downregulation of BMPR1A by miR-143-3p affected downstream signals, including Smad1/5/8 and its phosphorylation. As shown in Fig. 6C, overexpression of miR-143-3p significantly decreased the activity of phosphorylated Smad1/5/8, accompanied by an increase in the pro-apoptotic protein Bax and a decrease in the anti-apoptotic protein Bcl-2. Nevertheless, cotreatment with miR-143-3p mimic and BMPR1A expression plasmid could rescue the expression of p-Smad1/5/8 and anti-apoptotic protein Bcl-2 compared to miR-143-3p mimic alone. Moreover, overexpression of BMPR1A inhibited the expression of the pro-apoptotic protein Bax triggered by miR-143-3p. These results suggest that the BMPR1A/Smad1/5/8 signaling pathway mediates the role of miR-143-3p on cell apoptosis and proliferation in KGN cells.

## Discussion

Follicular fluid provides an important microenvironment for follicular development and oocyte maturation. The major components of follicular fluid are proteins, hormones, amino acids and metabolites^23^. Follicular fluid exosomes are new components in follicular fluid. It can deliver miRNAs, mRNAs and proteins involved in follicular development and oocyte maturation^24,25^. Previous studies have shown that miRNAs in exosomes derived from human follicular fluid play key roles in steroidogenesis and are closely associated with PCOS^26,27^. However, the mechanism of follicular fluid exosomal miRNAs in the pathological process of PCOS, especially in follicular development, is unclear.

In this study, FF-derived exosomes were isolated and characterized from patients with PCOS and healthy controls. We further identified the miRNA expression profiles in the exosomes derived from follicular fluid of PCOS and healthy control patients. Small RNA sequencing analysis showed that 20 miRNAs were differentially expressed between the PCOS FF-exosomes and the control group according to the *GRA001999* database of the National Genomics Data Center, among which miR-143-3p was enriched in PCOS-FF exosomes. Additionally, some studies have shown that miR-143-3p is involved in cell apoptosis^28,29^. Thus, we speculated that PCOS-FF exosomes might affect the apoptosis of granulosa cells by transferring miR-143-3p. In the present study, we found that overexpression of miR-143-3p significantly enhanced the apoptosis rate of GCs, accompanied by the inhibition of cell proliferation. Therefore, it is suggested that exosomal miR-143-3p in PCOS follicular fluid may play an important role in PCOS follicular dysplasia by affecting the biological functions of GCs.

Bone morphogenetic proteins (BMPs) belong to the transforming growth factor β (TGFβ) superfamily. Their biological effects are mediated by BMP-specific type I (BMPR1A and BMPR1B) and type II (BMPR2) serine/threonine kinase receptors as well as SMAD-related proteins^30^. BMPs are required for normal folliculogenesis in the ovary by regulating several key biological processes, including cell proliferation, differentiation, apoptosis, and steroidogenesis^21,31–33^. Thus, it is suggested that disruption of BMP/Smad signaling may be involved in folliculogenesis disorders. However, whether exosomal miR-143-3p regulates the function of granulosa cells by controlling BMPs or BMP receptors is still unclear. In this study, BMPR1A was predicted as a potential target of miR-143-3p by bioinformatics analysis, and luciferase reporter assays further verified the direct binding of miR-143-3p in the 3’UTR of BMPR1A in KGN cells. We further demonstrated that miR-143-3p promoted apoptosis and suppressed proliferation of KGN cells by directly targeting BMPR1A. BMPR1A mediates the miR-143-3p-regulated promotion of apoptosis and suppression of proliferation in granulosa cells. We also found that miR-143-3p-mediated cell apoptosis and proliferation in KGN cells were related to the phosphorylation of Smad1/5/8.

In conclusion, our research revealed for the first time that miR-143-3p-containing exosomes derived from PCOS follicular fluid promote granulosa cell apoptosis by targeting BMPR1A and blocking the Smad1/5/8 signaling pathway. Therefore, these findings provide novel insights into GC dysfunction in PCOS and suggest that exosomal-miR-143-3p derived from follicular fluid of PCOS patients may be a promising molecular target for the treatment of PCOS.

## Methods

### Clinical samples, ovarian stimulation

The clinical samples were obtained from 66 patients with PCOS and 80 healthy controls undergoing in vitro fertilization (IVF) at the Department of Reproductive Medicine Center, the Fourth of Shijiazhuang Maternity Hospital between April 2020 and September 2021. PCOS was diagnosed in strict accordance with the 2003 Rotterdam criteria, excluding Cushing’s syndrome, androgen secreting tumors, thyroid dysfunction, endometriosis, hyperprolactinemia and other endocrine diseases. All analyses performed in studies involving human samples were approved by the Clinical Ethics Review Board of the Obstetrics and Gynecology Hospital of Shijiazhuang (approval number: 20210080). All methods were performed in accordance with the relevant guidelines and regulatory methods section. All participants signed informed consent forms. Additionally, this study was conducted in accordance with the 1964 Declaration of Helsinki.

Follicular fluid (FF) was obtained from PCOS and healthy patients who underwent IVF-ET by controlled ovarian hyperstimulation (COH). On the second or third days of menstruation, a GnRH agonist (Ferring, Germany) was used, with the initial dose depending on the patient’s age, body mass index (BMI), basal hormone levels, and antral follicle count (AFC). After approximately 28 days, when the downregulation criteria were FSH < 5 mIU/mL, LH < 5 mIU/mL, E2 < 50 pg/mL, AFC approximately 5 mm and endometrial thickness < 5 mm, Gn was subcutaneously injected until the hCG day, and oocyte retrieval was performed by transvaginal ultrasound-guided aspiration after 36 h of hCG trigger.

### Isolation and identification of follicular fluid exosomes

Exosomes were isolated from healthy control patients (female tubal factors) and PCOS patients’ follicular fluids using the exoEasy Maxi Kit (Qiagen, Germany) according to the manufacturer’s instructions. Finally, the pellets containing exosomes were resuspended in XE buffer. The exosome protein content was quantified using the BCA Protein Assay Kit (Pierce™, Thermo Fisher Scientific, USA) and the absorbance measured at 562 nm was used to reflect the exosome protein concentration. Finally, freshly prepared exosomes were immediately used for experiments or were frozen at − 80 °C for further experimentation.

Characterization of FF-exosomes was confirmed by transmission electron microscopy (TEM): 5–10 μL exosomes were dropped onto carbon-coated copper grids at room temperature for 3–5 min, and then absorbed the excess liquid with absorbent paper. Then, 10 μL of 2% phosphotungstic acid was pipetted onto the grids for staining for 2–3 min, the excess fluid was removed, and the grid was dried at room temperature. Finally, the copper grid was detected under TEM at 80 kV (HITACHI). The exosome -specific marker TSG101 and EV-associated protein marker HSP70 were analysed by Western blotting.

### Isolation and culture of granulosa cells from follicular fluids

On the day of oocyte retrieval, the first tube of serum-free follicular fluid was collected, and then centrifuged at 2000*g* for 10 min. The supernatant was stored at −80 °C for further experiments. The cell pellets were resuspended in phosphate-buffered saline (PBS) and 1:1 added Ficoll solution, and then centrifuged for 15 min at 1500*g*. The cells at the interface were removed and washed twice with PBS, and the cell pellets were resuspended in DMEM/F-12 (HyClone, Logan, UT, USA) containing 10% fetal bovine serum (FBS) (Gibco, Carlsbad, CA, USA) supplemented with 100 U/mL penicillin and 100 μg/mL streptomycin at 37 °C in a 5% CO_2_ cell culture incubator.

### Exosome uptake assay

The purified exosomes were labeled with a PKH67 green Fluorescent Cell Liner Kit (Sigma–Aldrich, St. Louis, MO, USA) according to the manufacturer’s instructions. Briefly, 20 μg exosome-XE was added to 0.5 mL Dilution C, and 1 μL PKH67 was added to 0.5 mL Dilution C and then fully mixed and incubated with each other at room temperature for 3 min. Then, 1 mL 1% bovine serum albumin was added to neutralize the excess dye. Then, exosome-PKH67 was extracted with an exoEasy Maxi Kit following the manufacturer’s instructions (Qiagen, Germany). The labeled exosomes were then coincubated with 2 × 10^4^ primary GCs or 2 × 10^4^ KGN cells for 12 h. Then, the cells were fixed with 4% paraformaldehyde at room temperature for 10 min and washed twice with PBS, and the nuclei were stained with DAPI. Finally, the signal was observed under confocal microscope.

### RNA extraction, reverse transcription and RT-PCR analysis

Total RNA was extracted with TRIzol reagent (Invitrogen, Carlsbad, CA, USA), and then 1 μg of RNA was reverse-transcribed into cDNA. qRT-PCR was performed using the SYBR Premix Ex Taq kit in accordance with the manufacturer’s protocol. The exosomal RNA was extracted using the exoRNeasy Serum/Plasma Starter Kit (Qiagen, Germany). miRNA was then reverse-transcribed into cDNA by Bulge-Loop™ miRNA RT Primer (RiboBio Co., Ltd., Guangzhou, China). U6 small nuclear RNA was used as the internal reference for miRNAs. Ct values were indicated by using the 2^−ΔΔCt^ method.

To quantify the mRNA expression of the apoptosis-related genes Bax and Bcl-2, we used oligo d(T) 18 primers to reverse transcribe total RNA into cDNA. Then, qRT–PCR was performed by using SYBR Green dye and specific primers for Bax, Bcl-2 and β-actin. The primer sequences we used are listed in Table S1.

### Western blotting

SDS lysis buffer freshly mixed with a protease and phosphatase inhibitor cocktail (Thermo Scientific, Rockford, Cambridge, MA) was used to isolate proteins from cells. Western blotting assays were performed as described previously^34^. All the antibodies we used are listed in Table S2.

### miR-143-3p mimic and inhibitor transfection

The human ovarian granulosa-like tumor cell line KGN was transiently transfected with 50 nM miR-143-3p mimic, mimic NC, 100 nM miR-143-3p inhibitor and inhibitor NC according to the manufacturer’s instructions (RiboBio, Guangzhou, China) for 24 h or 48 h.

### Apoptosis and proliferation assay

Cell apoptosis Assays were conducted as described in the PE Annexin V Apoptosis Detection Kit (BD Biosciences, San Jose, CA, USA) according to the manufacturer’s instructions. The cells were pretreated with exosomes or miR-143-3p mimic or inhibitor (RiboBio Co., Ltd., Guangzhou, China), and 48 h after treatment, the cells were collected and diluted to a density of 1 × 10^5^, which was resuspended in binding buffer. Then, 5 μL of PE Annexin V and 7-AAD were introduced. The cells were incubated at room temperature in darkness for 15 min. Apoptotic cells were observed by flow cytometry (BD Biosciences, Franklin Lakes, NJ, USA).

The cell proliferation assay was conducted according to the manufacturer’s instructions. KGN cells were plated in 96-well plates at a concentration of 5000 cells/well. After 24 h, 48 h and 72 h of incubation, the cells were incubated with a 10 μl Cell Counting Kit-8 (CCK8) (Yeasen Biotech Co., Ltd. Shanghai, China) for another 3 h at 37 °C in a 5% CO_2_ cell culture incubator. Finally, the absorbance at 450 nm was measured by microplate reader.

### Vector construction and dual-luciferase reporter assay

MiRNAs that target BMPR1A were predicted by the online software program TargetScan Human 7.2. The full-length 3’UTR BMPR1A containing the predicted wild-type or mut-type miR-143-3p binding sites was amplified by PCR and then cloned into the PGL3-Basic reporter vector. PGL-3-BMPR1A-wt plasmids and PGL-3-BMPR1A-mut plasmids were cotransfected with miR-143-3p mimic into KGN cells along with the pRL-TK vector using Lipo2000 reagent (Invitrogen, Carlsbad, CA, USA). After 24 h of transfection, luciferase activity was determined using a dual-luciferase reporter assay system (Promega, Madison, WI, USA) according to the manufacturer’s protocol, and Renilla luciferase activity was used for normalization.

### Statistical analysis

All experiments were performed in the study as least two independent times each in duplicate. The measured data are presented as mean ± standard deviation. Clinical data were analysed by SPSS 17.0 software (SPSS Inc, Chicago, IL, USA). Quantitative data analysis was performed using GraphPad Prism software version 7.0. The differences in statistical significance between different treatments were evaluated by one-way ANOVA or Student’s t-test. *P* values lower than 0.05 were considered statistically significant.

### Ethics declaration

The study was approved by the Clinical Ethics Review Board of the Obstetrics and Gynecology Hospital of Shijiazhuang (Hebei, China), ethical approval number: 20210080.

## Supplementary Information

Supplementary Information.

## Data Availability

The datasets generated during and/or analysed during the current study are available from the corresponding author on reasonable request.

## Abbreviations

PCOS: Polycystic ovary syndrome
FF: Follicular fluid
GCs: Granulosa cells
Exos: Exosomes
TEM: Transmission electron microscope
IVF: In vitro fertilization
FSH: Follicle-stimulating hormone
E2: Estradiol
LH: Luteinizing hormone
PRL: Prolactin
T: Testosterone
MII: Metaphase II oocytes
