# Supplementary material for: Exosomal miR-143-3p derived from follicular fluid promotes granulosa cell apoptosis by targeting BMPR1A in polycystic ovary syndrome
Source: Sci Rep. 2022 Mar 14;12:4359. doi: 10.1038/s41598-022-08423-6 (PMC8921316; doi:10.1038/s41598-022-08423-6)
Supplement: Supplementary file 1 — Supplementary Information. [file 41598_2022_8423_MOESM1_ESM.pdf]

Supplementary Information

Exosomal miR-143-3p derived from follicular fluid promotes granulosa cell apoptosis by targeting BMPR1A in polycystic ovary syndrome

Yuanyuan Zhao[1,2], Shuhong Pan [1,2], Yunying Li[1,2], XiaohuaWu[1,2]*

[1]Center for Reproductive Medicine, the Fourth Hospital of Shijiazhuang(Gynecology and Obstetrics Hospital Affiliated to Hebei Medical University), 206 East Zhongshan Road, Shijiazhuang050011, Hebei, China;

[2]The institute of Reproductive Health and Infertility, Shijiazhuang050011, Hebei, China;

*Corresponding Author: Xiaohua Wu, Center for Reproductive Medicine, The Fourth Hospital of Shijiazhuang(Gynecology and Obstetrics Hospital Affiliated to Hebei Medical University), 206 East Zhong-shan Road, Chang-An District, Shijiazhuang050011, Hebei, China. E-mail: wuxiaohua1965@163.com.

Supplementary Figure S1

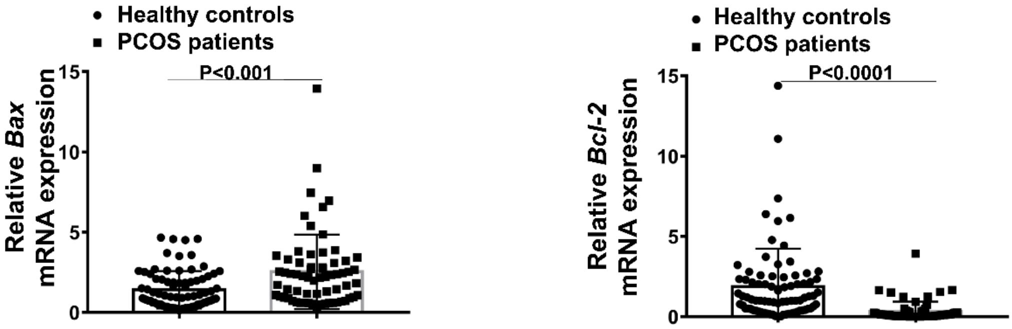

Supplementary Figure S1. Enhancement of Bax mRNA expression and inhibition of Bcl-2 mRNA expression in primary GCs of patients with PCOS

Quantitative PCR analysis of expression levels of apoptosis related genes in primary GCs of healthy controls (n=80) and PCOS group (n=66). **P* <0.05, ***P* <0.01, ****P* <0.001, All results are presented as the mean ± SD.

Supplementary Figure S2

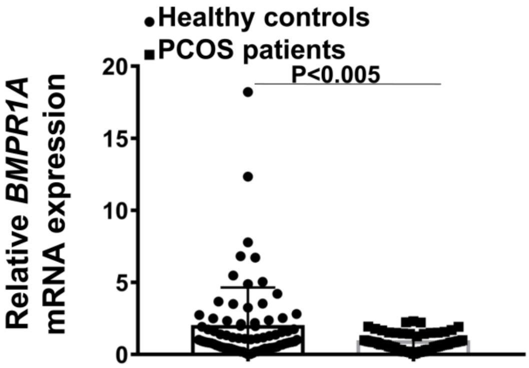

Supplementary Figure S2. Inhibition of BMPR1A mRNA expression in primary GCs of patients with PCOS

Quantitative PCR analysis of expression levels of BMPR1A in primary GCs of healthy controls (n=80) and PCOS group (n=66). **P* <0.05, ***P* <0.01, ****P* <0.001, All results are presented as the mean ± SD.

Supplementary Figure S3

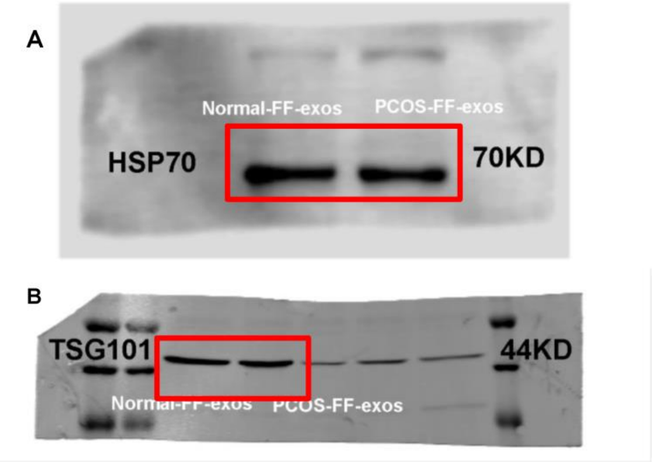

Supplementary Figure S3. Full scans of original blots for data in Figure 1B.

Whole gel images of Western blots of HSP70 (A) and TSG101 (B), which are typical exosome markers in Figure 1B. Both blots represented unprocessed original image data, and the cropped area is shown in Figure 1B.

Supplementary Figure S4

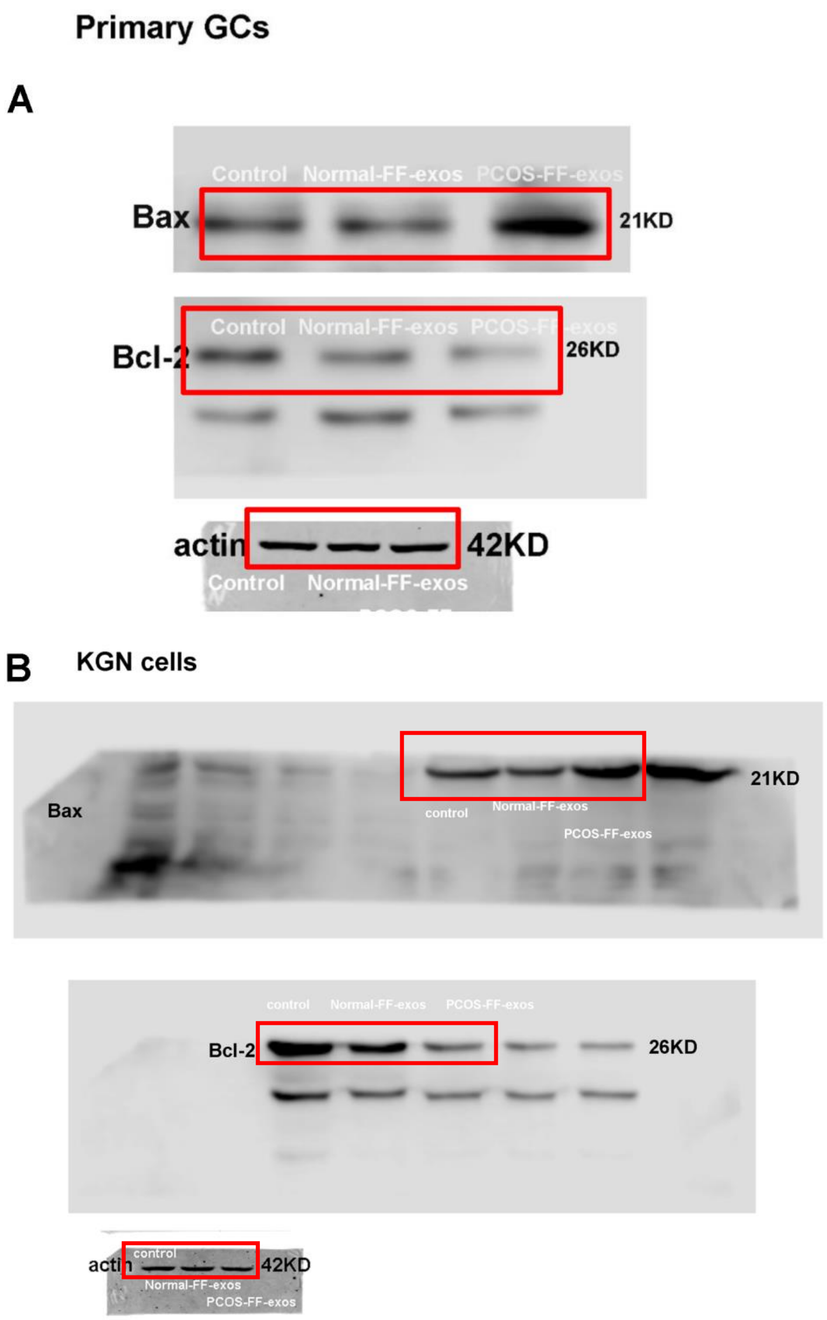

Supplementary Figure S4. Full scans of original blots for data in Figure 2B.

Full-length Western blots of Bax, Bcl-2 and β-actin in primary GCs (A) and in KGN cells(B) in Figure 2B. The membrane blots represented unprocessed original image data, and the cropped area is shown in Figure 2B.

Supplementary Figure S5

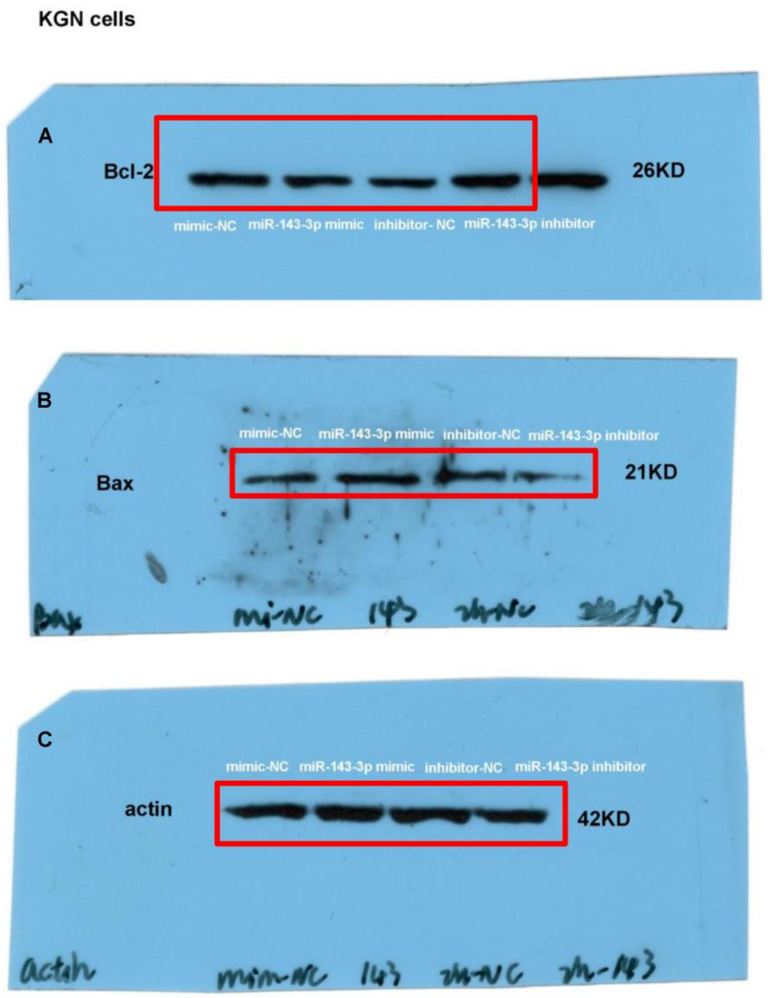

Supplementary Figure S5. Full scans of original blots for data in Figure 4B.

Full-length Western blots of Bcl-2 (A), Bax (B) and β-actin (C) in KGN cells in Figure 4B. The membrane blots represented unprocessed original image data, and the cropped area is shown in Figure 4B.

Supplementary Figure S6

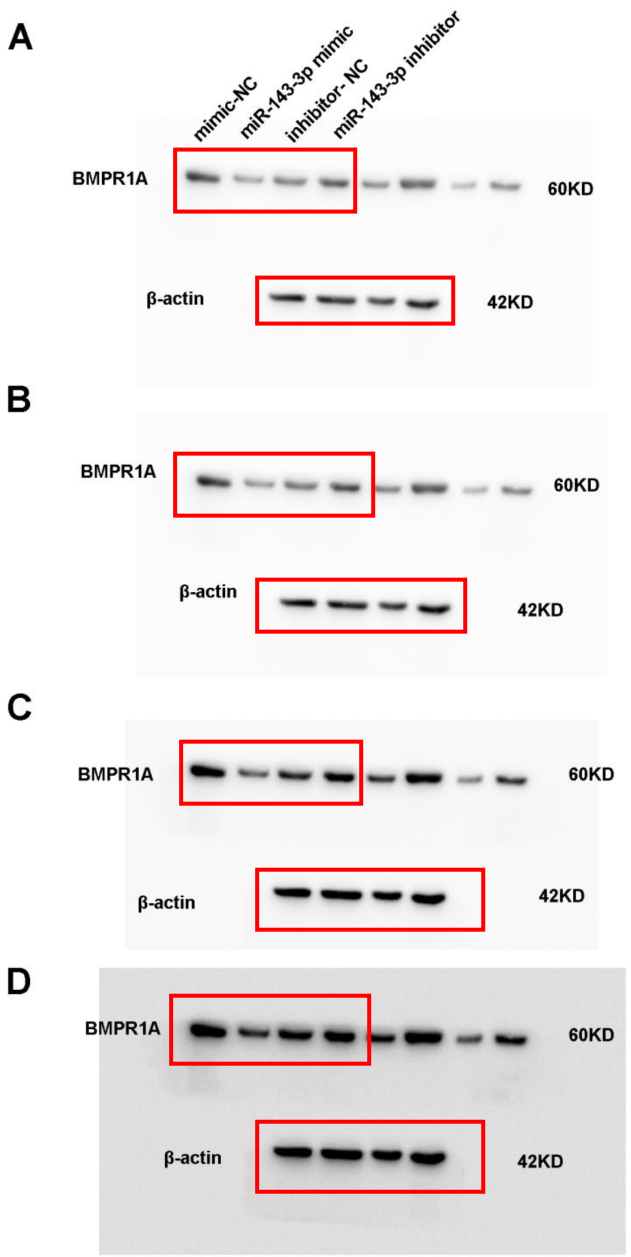

Supplementary Figure S6. Full scans of original blots for data in Figure 5D.

Whole gel images for Western blots of BMPR1A and β-actin in Figure 5D, the membrane represented unprocessed original image data, A-D indicated that the different exposure intensities of the strips.

Supplementary Figure S7

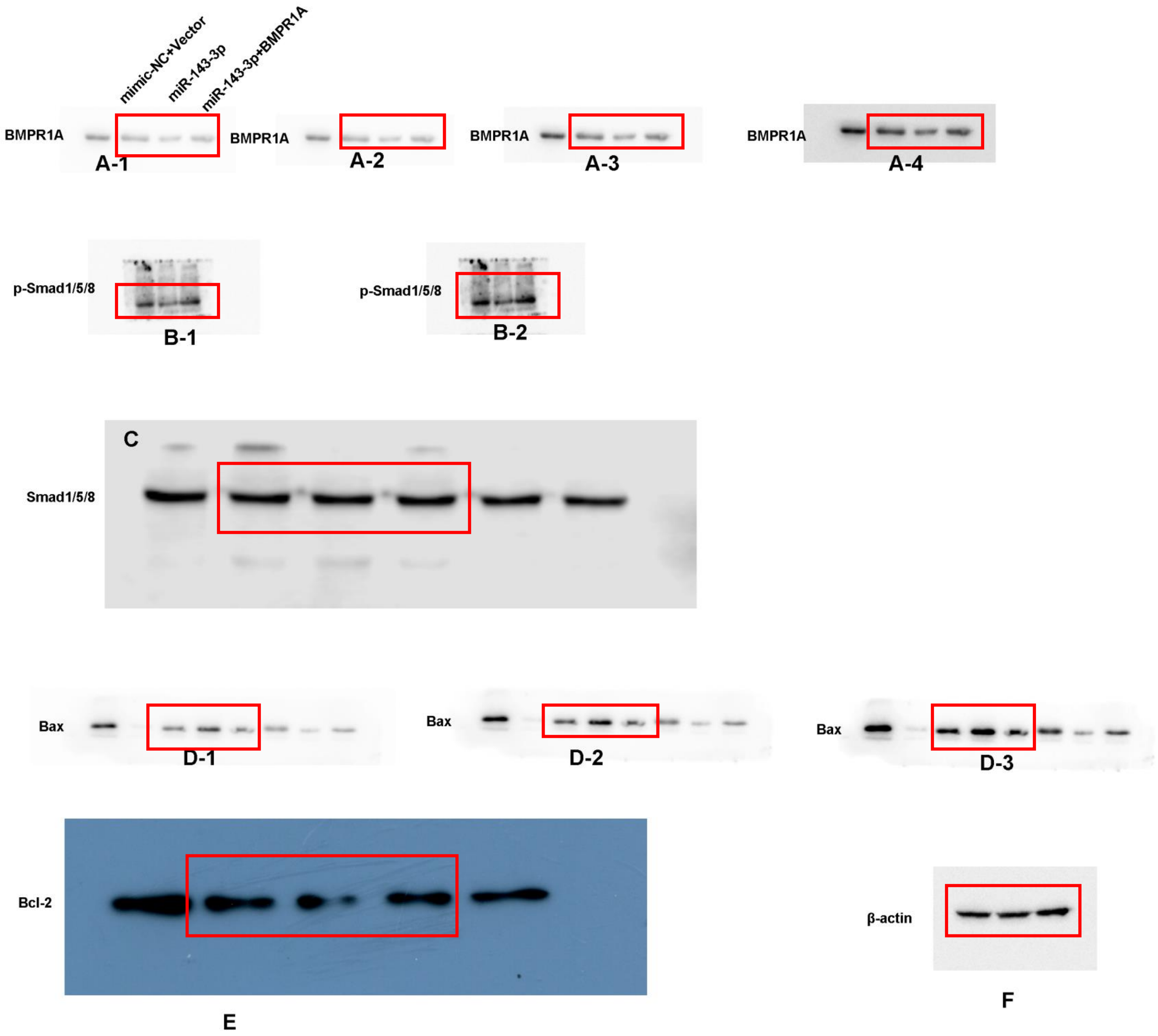

Supplementary Figure S7. Full scans of original blots for data in Figure 6C

Whole gel images for Western blots of BMPR1A, p-Smad1/5/8, Smad1/5/8, Bax, Bcl-2 and β-actin in Figure 6C, the membrane represented unprocessed original image data, A-1 to A-4 indicated that the different exposure intensities of BMPR1A; B-1 and

B-2 indicated that the different exposure intensities of p-Smad1/5/8; D-1 to D-3 indicated that the different exposure intensities of Bax in KGN cells treated with miR-143-3p mimic and/or BMPR1A-expressing plasmid.

Supplementary Table S1. Primer sequences for Q-PCR

Gene	Forward primer	Reverse primer
Bax	TCAGGATGCGTCCACCAAGAAG	TGTGTCCACGGCGGCAATCATC
Bcl-2	ATCGCCCTGTGGATGACTGAGT	GCCAGGAGAAATCAAACAGAGGC
β-actin	CAGAGCAAGAGAGGCATCC	CTGGGGTGTTGAAGGTCTC

Supplementary Table S2. Antibodies used for Western blotting

Antibody name	Company, catalog number	Concentration of antibodies used
HSP70	Santa, sc-24	1:1000
TSG101	Abcam, ab125011	1:1000
BAX	ZEN BIO, 200958	1:1000
Bcl-2	ZEN BIO, 381702	1:500
Smad1/5/8	Abcam, ab80255	1:1000
p-Smad1/5/8	CST, #13820	1:1000
BMPR1A	Abclonal, A1816	1:1000
β-actin	Sigma-aldrich, A5441	1:10000
